# Developing a Self-Administered Decision Aid for Fecal Immunochemical Test–Based Colorectal Cancer Screening Tailored to Citizens With Lower Educational Attainment: Qualitative Study

**DOI:** 10.2196/formative.9696

**Published:** 2018-05-22

**Authors:** Pernille Gabel, Pia Kirkegaard, Mette Bach Larsen, Adrian Edwards, Berit Andersen

**Affiliations:** ^1^ Department of Public Health Programmes Randers Regional Hospital Central Denmark Region Randers NØ Denmark; ^2^ Department of Clinical Medicine Faculty of Health Aarhus University Aarhus Denmark; ^3^ Division of Population Medicine School of Medicine Cardiff University Cardiff United Kingdom

**Keywords:** colorectal neoplasms, mass screening, decision support techniques, socioeconomic factors, qualitative research

## Abstract

**Background:**

Citizens with lower educational attainments (EA) take up colorectal cancer screening to a lesser degree, and more seldom read and understand conventional screening information than citizens with average EAs. The information needs of citizens with lower EA are diverse, however, with preferences ranging from wanting clear recommendations to seeking detailed information about screening. Decision aids have been developed to support citizens with lower EA in making informed decisions about colorectal cancer screening participation, but none embrace diverse information needs.

**Objective:**

The aim of this study was to develop a self-administered decision aid for participation in fecal immunochemical test–based colorectal cancer screening. The decision aid should be tailored to citizens with lower EA and should embrace diverse information needs.

**Methods:**

The Web-based decision aid was developed according to an international development framework, with specific steps for designing, alpha testing, peer reviewing, and beta testing the decision aid. In the design phase, a prototype of the decision aid was developed based on previous studies about the information needs of lower EA citizens and the International Patient Decision Aid Standards guidelines. Alpha testing was conducted using focus group interviews and email correspondence. Peer review was conducted using email correspondence. Both tests included both lower EA citizens and health care professionals. The beta testing was conducted using telephone interviews with citizens with lower EA. Data were analyzed using thematic analysis.

**Results:**

The developed decision aid presented information in steps, allowing citizens to read as much or as little as wanted. Values clarification questions were included after each section of information, and answers were summarized in a “choice-indicator” on the last page, guiding the citizens toward a decision about screening participation. Statistics were presented in both natural frequencies, absolute risk formats and graphically. The citizens easily and intuitively navigated around the final version of the decision aid and stated that they felt encouraged to think about the benefits and harms of colorectal cancer screening without being overloaded with information. They found the decision aid easy to understand and the text of suitable length. The health care professionals agreed with the citizens on most parts; however, concerns were raised about the length and readability of the text.

**Conclusions:**

We have developed a self-administered decision aid presenting information in steps. We involved both citizens and health care professionals to target the decision aid for citizens with lower EA. This decision aid represents a new way of communicating detailed information and may be able to enhance informed choices about colorectal cancer screening participation among citizens with lower EA.

## Introduction

Colorectal cancer (CRC) has particularly high mortality among disadvantaged groups, including those with low educational attainment (EA) [1,2]. A US study observed that the mortality rate of those primarily with higher EA decreased between the years 1993 and 2001, whereas it increased for those with lower EA [3].

Screening using the guaiac fecal occult blood test (gFOBT) may reduce both CRC incidence and mortality by removing precancerous adenomas and detecting the earlier stage CRC [4]. Recent studies have determined that the fecal immunochemical test (FIT) is superior to gFOBT in detecting CRC [5-7], and hence, FIT has been implemented in an increasing amount of screening programs worldwide [8-10]. In addition to screening benefits, screening harms, such as risk of overdiagnosis and risk associated with invasive procedures, also exist, thereby making participation in screening beneficial for some individuals and more or less harmful for others [4]. Hence, the decision to take up CRC screening is a preference-sensitive choice, that is, a choice that should be based on adequate knowledge about screening and reflect personal values [11,12].

Deprived populations tend to participate less in CRC screening than others [13], and this may reflect a lack of screening knowledge as well as social barriers [14]. Health authorities in countries offering CRC screening provide citizens with information on CRC screening, but a Dutch study has shown that conventional information material, although of high quality and with few unique content words per paragraph, might be overwhelming for citizens with low health literacy and lower EA [15]. The study showed that citizens with lower EA tend to read only headings and look at pictures [15].

Decision aids (DAs) are evidence based and aim to support citizens in making specific choices about health-related issues. In general, they improve knowledge, decrease decisional conflict, and increase the proportion of citizens being active in the decision-making process [16]. Several DAs have been developed for CRC screening [17-21]. These DAs must be self-administered, as the citizens receive the screening-kit by mail, obtain the sample at home, and mail it directly to the laboratory for analysis. In general, these DAs increase citizens’ knowledge of CRC and CRC screening, enhance informed decision-making, and decrease decisional conflict [17,18,20,21]. However, the effect of the DAs on the participation rate is not conclusive [17-19,21].

An increasing amount of information from health authorities occurs via eHealth and mHealth (electronic- or mobile-based health) solutions. Email, text messages, and various Web services are used to provide information and to communicate scheduled appointments, reminders, test results, etc. EHealth has the same effect on health care appointment attendance, screening uptake, and general well-being as the traditional conventional mailing system and telephone calls, but it is cheaper and faster [22,23].

Few DAs are also available in eHealth formats (Web pages, apps, etc) [24]. Web-based DAs have advantages in easy accessibility and the potential for broadened reach and regular updates. However, regardless of the format, the DA must be developed according to the targeted citizens’ information needs [25]. Citizens with lower EA have diverse information needs [26], but few DAs have been specifically tailored to citizens with lower EA [17,27-29] and none have been developed, embracing diverse information needs in CRC screening.

The aim of this study was to develop and field test a Web-based, self-administered DA for FIT-based CRC screening, embracing diverse information needs tailored to 50- to 74-year-old citizens with lower EA.

## Methods

### The Danish Setting

The implementation of population-based CRC screening in the Danish health care system began in 2014, and it was fully implemented in 2018 from when eligible 50-74-year-old Danish citizens will be invited biennially to CRC screening using FIT. The invitation contains a screening kit for obtaining a fecal sample to be submitted directly to the laboratory for analysis. If a sample is not submitted within 6 weeks, a digital reminder is sent.

In Denmark, secure digital communication with authorities is mandatory [30], although disabled citizens can be exempt and continue to receive conventional mail [30]. In July 2017, 8.7% of the Danish population aged 45-74 years was exempt from digital communication [31]. Thus, CRC screening communication occurs mainly via secure digital mails, except for invitation letters containing a screening kit, and positive screening results that include an invitation to follow up colonoscopy and medication for bowel preparation.

### Planning the Development

In the context of mandatory digital communication in Denmark, we chose to develop a digital DA, using the validated and internationally accepted framework proposed by Coulter et al [32], on the basis of the International Patient Decision Aid Standards (IPDAS) [33]. This framework describes the development process in 5 steps: (1) the scoping of the DA, (2) the formation of the steering group (preferably multidisciplinary), (3) the design phase, (4) alpha testing (user testing), and (5) beta testing (field testing). This method also corresponds to previously proposed frameworks for the development of eHealth solutions of high reliability, usefulness, and quality [34].

**Figure 1:**
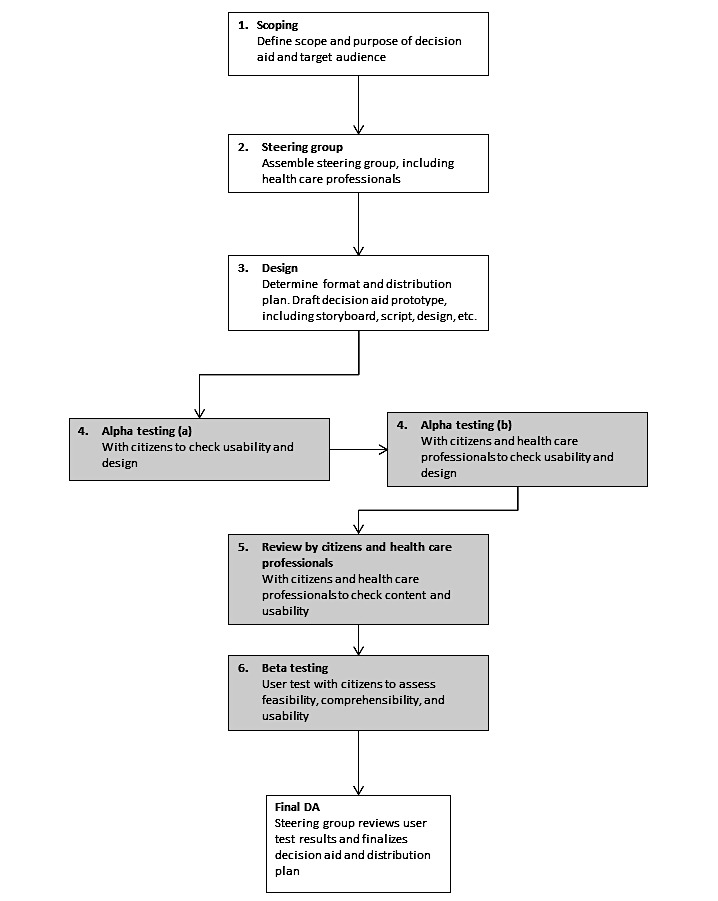
Framework for the decision aid development. Adapted from Coulter et al [32]. Gray boxes indicate involvement of a citizen or health care professional. DA: decision aid.

Figure 1 depicts the development process for the DA (adapted from Coulter et al [32]). Steps 1 and 2 were carried out according to the framework. In the design phase (Step 3), a prototype of the DA was drafted, based on the citizens’ information needs and preferred format, as described in a previous study, ranging from preferences to receive a clear recommendation with a minimum of information to desires for a detailed information and the opportunity to make a highly informed decision [26]. In that study, most participants agreed that information about CRC symptoms, benefits and harms of screening, and instructions to perform the FIT test were relevant information, and information should be presented in bullet points or as flowcharts, using absolute numbers. The DA should be accessible via the Internet. Information about colonoscopy, however, was requested only by those wanting detailed information [26]. In this study, the specification of the DA prototype adhered to the IPDAS instrument and checklist. It was based on the 4 domains of content: (1) providing information, (2) presenting probabilities, (3) including methods for values clarification and expression, and (4) recommending support [25,35]. Furthermore, as developed by Clerehan et al [36] and validated by Hirsh et al [37], the content was evaluated by using the 9 items of the evaluative linguistic framework: (1) generic structure, (2) rhetorical elements, (3) meta-discourse, (4) headings, (5) factual content, (6) technicality, (7) lexical density (average number of content words per clause), (8) writer and reader relationship, and (9) format. Throughout the development phase, texts were kept as short as possible while taking the information needs into account. The lexical density was assessed for the final DA, as described in the evaluative linguistic framework [36]. We chose to develop a Web-based DA, presenting information in steps, and thereby embracing diverse information preferences. The DA was an interactive Web page with no specific outcome or product.

### Participants

Citizens with lower EA were residents of the Central Denmark Region aged 50-74 years. They were recruited for Steps 4-6 via an external professional recruitment company [38]. Lower EA is defined according to the United Nations Educational, Scientific, and Cultural Organization classification of basic education (ISCED 2011) Levels 1-2 [39], which is equivalent to less than 10 years of education in Denmark, corresponding to 24% of the population in the targeted age group [40]. The recruitment company recruited citizens from an existing panel of citizens who voluntarily signed up to receive regular Internet-based surveys on various health and nonhealth topics. The Internet skills of the participants were not measured, but skills at or above average were assumed, due to regular Internet-based survey activity. At recruitment, the citizens agreed to take part in either a focus group interview or in a telephone interview. The citizens who accepted to take part in the focus group or the review (Step 5) were told that they would receive a gift (of value US$ 80) as a token of the appreciation for their time. Furthermore, the travel expenses would be covered. Health care professionals were recruited via the professional network surrounding the Danish National CRC screening program in the Central Denmark Region. Both general practitioners (GPs) and colonoscopists with responsibilities for CRC screening were recruited.

### Alpha and Beta Testing

For the first alpha testing (Step 4a), we conducted focus group interviews with citizens to evaluate the design and usability of the prototype DA. According to Coulter et al [32], this step should also evaluate comprehensibility; however, as the citizens’ information needs and their preferred figure and chart representations were already described [26], we deferred this evaluation to Step 5. For the second alpha testing (Step 4b), we conducted email correspondence with citizens and health care professionals, exploring usability, acceptability, and design.

The review (Step 5), particularly focusing on content and readability, involved email correspondences with citizens and health care professionals not previously involved in the development process. Thus, we included more health care professionals and citizens in the development process than would have been the case with only the steering group conducting the review, as proposed by Coulter et al [32].

The beta testing (Step 6), including semistructured telephone interviews with citizens, examined feasibility, comprehensibility, and usability. No clinicians were involved in this step as the decision to take up CRC screening is usually made by citizens alone, without contacting health care professionals.

### Data Collection

On the basis of the themes for the specific development steps, semistructured interview guides were developed (Multimedia Appendices 1-4). During the focus group interview (Step 4a), the citizens read the DA without any introduction. They were asked to think aloud about any immediate impressions of the DA. After this session, the semistructured interview guide (Multimedia Appendix 1) was used for a discussion of the DA. The first author (PG) and a coauthor (PK) were present, and both observed and made notes, which were later compared. In email correspondences (Steps 4b and 5), questions guiding the respondents to the focus of the evaluation were sent to the citizens and health care professionals (Multimedia Appendices 2 and 3). The telephone interviews (Step 6) were based on a semistructured interview guide as well. Both open-ended and categorical questions were asked in the telephone interviews (Multimedia Appendix 4).

### Analysis

All data from meetings, email correspondences, and interviews were divided into specific datasets corresponding to each step of the development process. A thematic analysis was conducted for each dataset focusing on readability, usability, comprehensibility, and feasibility [41]. Data coding was done by the first author (PG) and subsequently discussed with the coauthors.

## Results

### Prototype (Step 3)

The development of the prototype was based on the IPDAS guideline and checklist as well as the evaluative linguistic framework [25,35,36] A simple and appealingly designed DA with only 3 different colors was developed by an external Web agency [42]. The texts were kept as short as possible with the font size 12. The information was presented in a plain language, with a minimum of medical terms used. A site map was provided in the left-side margin, and help options and contact information were provided in the right-side margin. At the bottom of each page, a status bar showed a user’s progress through the DA content (7 steps). There were 16 pages in total. On the first page, the purpose of the DA was explicitly stated, thereby also emphasizing the sender’s role as informant and the reader’s role as an active decision maker.

**Figure 2:**
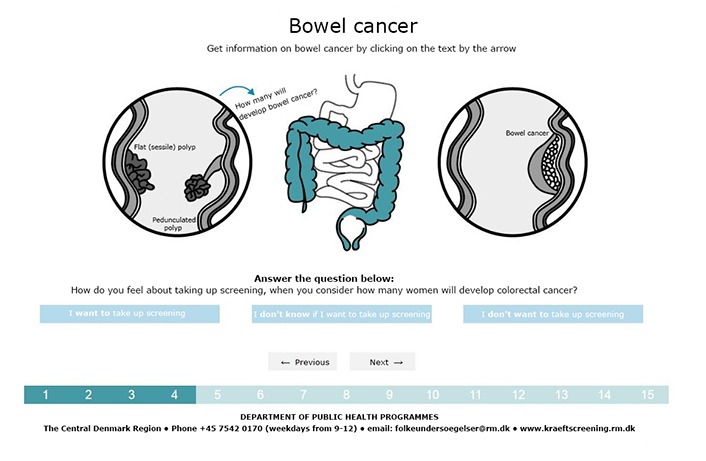
Page from the final decision aid.

Each page consisted of a heading, a figure, and a values clarification question (Figure 2). A pop-up with additional information was accessible via a link in the figure. Furthermore, most pop-ups had a *read-more* option with detailed information. In this way, information was presented in steps, allowing the reader to read as much or as little as desired. The relevant subjects were presented in an intuitive order, and the function of each clause was underpinned as informative by writing in general terms, or as instructive by speaking directly to the reader (using singular personal pronouns).

Information in the DA was selected according to the IPDAS instrument dimensions (information, probabilities, values, decision guidance, development, evidence, disclosure, plain language, evaluation, and test), addressing all content dimensions. Development and evaluation are addressed in this paper [25]. Information was derived from both the Danish Colorectal Cancer Screening Database [43] (participation rates, positive FIT, etc), Statistics Denmark [44] (Central Denmark Region population of 50- to 74-year-old citizens), a systematic review [45] (general effect of CRC screening), and NORDCAN (CRC prevalence, incidence, and mortality) [46]. Two versions were developed, 1 for men and 1 for women, as incidence and mortality rates differ according to sex [46].

**Figure 3:**
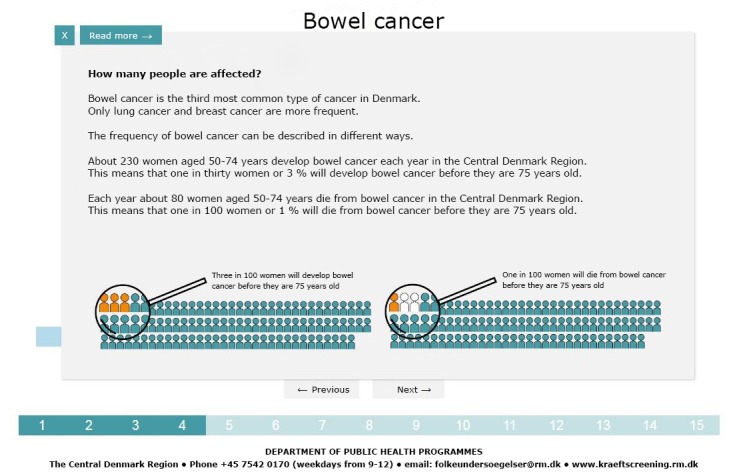
Pop-up from the final decision aid.

**Figure 4:**
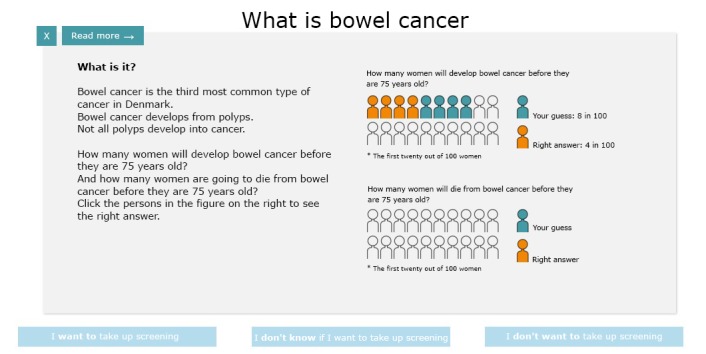
Interactive pictograms in prototype of decision aid.

All estimates were presented in both natural frequencies and absolute risk formats, sometimes also in pictograms and charts (Figure 3). The DA encouraged reflection on facts by providing interactive pictograms, in which the proportions were to be guessed, immediately followed by a presentation of the correct proportion (Figure 4). The values clarification questions encouraged reflection at each step on personal values. On the last page, the DA provided a *choice indicator* with an arrow pointing toward “Want to participate”, “Don’t want to participate”, or somewhere in between. Along with the indicator, a printable list was provided, presenting the answers given to the values clarification questions. The DA encouraged users to think about participation in screening and to talk to a doctor or relatives about the decision, if necessary.

### Alpha Testing With Citizens (Step 4a)

A total 5 out of 6 citizens accepted to participate in the planned focus group, of whom 3 did not attend the meeting in November 2016 and the remaining 2 citizens evaluated the DA.

In general, the citizens appreciated the initiative:

Finally someone talks to us as citizens, instead of just talking to each other as experts.

Female citizen: 66 years

They easily navigated around the pages and intuitively knew how to do this:

It’s easy to press read more and to exit by clicking the X in the corner.

Female citizen: 66 years

They found the DA useful and would recommend it to friends and family if it were available.

The design was accepted as appropriate:

I like the set-up, the design and the colors. Not too clinical, but not too frisky either – it’s official looking, and appealing.

Male citizen: 71 years

The interactive pictograms were, however, difficult to understand, and “Factual knowledge instead of guesswork” (female citizen: 66 years) was preferred.

These findings from Step 4a were discussed in the steering group and the pictograms were amended to be static and no longer interactive.

### Alpha Testing With Health Care Professionals and Citizens (Step 4b)

In December 2016, the revised DA was sent to 2 health care professionals (a GP and a colonoscopist) and the 2 citizens from the citizen alpha testing (Step 4a). Usability and design were evaluated via emails.

In general, both citizens and health care professionals found the DA “extremely relevant” (colonoscopist). The citizens found the information “of suitable length…without it being too much” (male citizen: 71 years), whereas the health care professionals found that “the amount of text in the *read more* pop-ups seems large and could be difficult to understand for non-professionals” (colonoscopist).

Both citizens and health care professionals found the links that provided the pop-ups a little difficult to use, as the text stated to:

...click on the text...when in fact, it is the blue arrow you have to click on.

GP

Following this feedback, the texts in the pop-ups were redrafted to a plainer language, preserving the content. Furthermore, both text and arrows were activated as links for the pop-ups.

### Peer Review (Step 5)

For the peer review, 2 health care professionals (a GP—different from the one in Step 4b—and a nurse conducting colonoscopies related to screening) and 3 citizens were recruited. In December 2016, these 5 reviewers received an email containing a link to the DA, followed by telephone interviews.

Due to some technical difficulties, 1 citizen and 1 health care professional (nurse) could not review the DA.

The GP and the 2 remaining citizens approved the content. It is “good information material that is easy to understand” (female citizen: 66 years) and with “an appropriate amount of information” written in “a good readability index” (male citizen: 59 years). However, at some points, the text was felt to be on a “professional and technical level,” and contained “a lot of numbers and estimates” (GP).

The citizens found the DA “intuitive to use” (female citizen: 66 years). They would “definitely use it” (male citizen: 59 years) and “recommend it to others” (female citizen: 66 years).

Following this review, some passages in the pop-up texts were further revised to plain language and compatibility problems were resolved.

### Beta Testing (Step 6)

For beta testing, 21 citizens were recruited, of whom 20 participated. This was followed by a telephone interview, examining feasibility, usability, and comprehensibility. The included citizens represented both citizens with lower EA opposed to screening and those who were proscreening. Furthermore, both citizens with limited computer skills and citizens with average or excellent computer skills, and higher and lower incomes, were represented. Different occupational status was also represented: full time occupation, citizens who were retired, including some with early retirements. Most citizens stated this information during the telephone interview (Multimedia Appendix 4). However, data were not systematically collected.

In general, the citizens appreciated the design; they found it appropriate with “light pages and nice and simple figures, manageable and formal” (female citizen: 58 years). The content was also appreciated, and they found the DA “easy to read and comprehend” (female citizen: 66 years). A few expressed that there was a “tendency for too much information, it can be confusing” (female citizen: 58 years), and “I’m afraid many people will skip great parts of this” (male citizen: 64 years).

Most citizens spent less than 15 min going through the DA, and agreed that a link in an email would be a feasible way to access the DA. The values clarification questions were regarded as useful: “They are fine, they make you think” (female: 57 years) and “they are easy to comprehend” (female: 60 years). Most people felt encouraged to think about benefits and harms while reading the DA. On the basis of this user testing, minor revisions were made, primarily proof reading of text and setting up the online domain and hosting for the DA.

### Final Decision Aid

**Figure 5:**
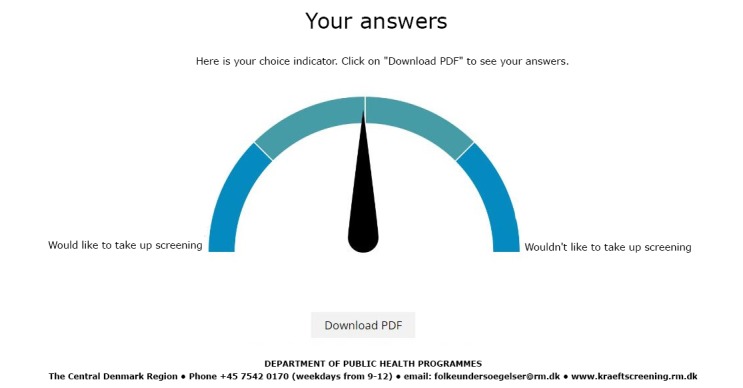
Choice indicator from the last page of the final decision aid.

The final DA was an interactive Web page. It consisted of 7 steps (15 pages in total). Each page contained a values clarification question and a figure or chart with links to pop-up text (Figure 2). The lexical density is generally 1.5 to 2 in the spoken language and 3 to 6 in the written language [36]. For the pop-ups in the original (Danish) DA, the lexical density was 3.3 (ie, the lower end of the written language). Most pop-ups (Figure 3) had a *read more* function, with a lexical density of 4.2, which is medium for the written language. On the last page, citizens were presented with the choice indicator (Figure 5) and the opportunity to print out their answers to the values clarification questions. The DA is available (in Danish) by contacting the authors. (Figures 2-5 are English translations of the original versions.)

## Discussion

### Principal Findings

We developed a self-administered DA for FIT-based CRC screening, embracing diverse information needs among citizens with lower EA. The initial prototype contained interactive elements (Figure 4), but these features were dismissed and removed in the final version. The remaining parts of the DA underwent minor revisions throughout the process, and citizens and health professionals accepted the design of the final DA as appropriate, official, and appealing. They appreciated the simplicity of the figures and the light colors. The content was considered relevant, and the citizens found it of suitable length without information overload. The health professionals, on the other hand, assessed it to be rather long and potentially difficult to understand for laypersons. The presentation of both absolute risks and natural frequency formats and the plain language were found comprehensible. Most citizens stated that they read only selected paragraphs of the DA. Most of them said that they would use the DA and recommend it to others.

### Strengths and Limitations

We followed a predefined framework for the development as proposed by Coulter et al [32]. However, as the developed DA is a self-administered DA not intended to be used by health care professionals, no (beta) user testing was done with health care professionals. The diverse information needs in citizens with lower EA as described by Kirkegaard et al [26] prompted the presentation of information in steps. Furthermore, the stepwise development of the DA made it possible to include a wide range of citizens and health care professionals and to use different ways of communication. Email correspondences and telephone interviews were convenient for the citizens to comment on the DA. Email correspondence was chosen to provide as much liberty as possible for the responses of health care professionals and citizens. According to a previous research, asynchronous email interviewing is an acceptable alternative to telephone interviews [47,48], also among citizens older than 65 years [49]. Email interviewing is cost-saving because less time is spent in participant transportation and data transcription. Furthermore, the email responses are often more deliberate and reflective in fewer words due to the respondents’ opportunity to edit before pressing send. The anonymity adds to the strengths of email interviewing because personal or complex subjects are more easily discussed. However, email interviewing requires more explicit questions, and caution is required because no facial expressions or personal interactions are observed in these interviews [47,49]. The face-to-face meeting provided an opportunity to observe the citizens going through the DA, and the citizens supplemented each other in the subsequent conversation about the DA. The use of a framework and previous findings have ensured a DA truly aimed at the targeted population, containing the most relevant and accessible information.

The citizens in this study were recruited from an existing citizen panel. Hence, they are likely more accustomed to using the Internet and more engaged in surveys than the rest of the population. This should be taken into consideration when transferring the results of this study to the general lower EA population because the most disadvantaged citizens may be the ones who experience most difficulties using the DA. However, some citizens who stated that they did not think of themselves as Internet knowledgeable and citizens who stated that they had less favorable attitudes toward CRC screening were recruited. Hence, we feel that the diversity of the population was represented to some degree in the study population.

The fact that only 2 citizens took part in Step 4a (the face-to-face meeting, planned as a focus group interview) might have compromised the generalizability of the feedback given during the meeting [50]. However, we consider that this is balanced by the comprehensive data collection opportunities in the following steps.

Technical problems were experienced during the alpha testing, and the citizens needed to start again with the DA several times. Both citizens in the face-to-face interview stated that they felt they had to hurry and would have spent longer reading it if they had been at home. Even though the content evaluation in this step might have been compromised somewhat, the technical problems helped us make technical adjustments, making the DA accessible from almost all types of electronic devices and Internet browsers.

### Interpretation of Results

Health care professionals generally expected the citizens to find the DA long and more difficult to understand than was reported by the citizens. This may be due to several factors. First, previous studies have shown that doctors are poor judges of their patients’ health beliefs [51] and priorities [52] when it comes to trade-offs over different treatment options. Second, the treatments doctors recommend for patients are often different from those they would choose if they were a patient, indicating that the counseling role is different from the patient role [53].

Citizens with lower EA often have lower levels of health literacy [54], and hence, they might experience difficulties reading and understanding health care information [15]. The length of the DA may, therefore, be at odds with its intended target audience of citizens with lower EA. The stepwise presentation of data in our DA may, however, have contributed to its readability and could explain why citizens in our study did not report information overload.

According to the IPDAS guidelines, DAs should have a values clarification exercise in some form [33]. In general, values clarification methods increase citizens’ attention to benefits and harms, and they are considered useful [55]. However, in this Danish setting, the paper format of the values clarification exercise was considered inapplicable [26]. In this study, the citizens liked the exercises, indicating that the format of the exercises might influence the acceptance and usability of the values clarification methods.

The DA was distributed via email because most citizens are expected to use eHealth solutions, as digital communication is mandatory [30]. However, eHealth solutions are less commonly used by citizens with lower EA [56,57]. According to Norman et al [58], eHealth literacy is an important skill to use eHealth solutions; eHealth literacy is defined as “the ability to seek, find, understand, and appraise health information from electronic sources and apply the knowledge gained to addressing or solving a health problem” [58]. EHealth literacy decreases with increasing age and with lower EA [59]. We sought to avoid exacerbating social inequality by using lay language and unique, easy to use Internet features in this newly developed DA.

A DA aims to give citizens enough information to make them feel they can make an informed choice about screening participation. This is important as CRC screening participation is a preference-sensitive choice [60]. Seeking to provide citizens with sufficient information, conventional information material contains detailed information about CRC and CRC screening. This might increase the existing social gradient in CRC screening because citizens with lower levels of health literacy are likely to read and understand these conventional information materials to a lesser degree [54]. For those citizens with lower EA who prefer a clear recommendation about screening rather than detailed information [26], there are questions about whether detailed information material is the best way of informing these citizens about CRC screening. However, citizens with preferences for detailed information should be able to access this. By providing information in a stepwise manner, we have sought to tailor the information to the needs of the individual citizen in the population, thereby potentially decreasing the social gradient in utilization of CRC screening information.

### Implications for Practice

The development of this self-administered DA may prove to be a new method of communicating detailed information about CRC screening to citizens with lower EA, with built-in flexibility to avoid information overload. The effect of the DA on knowledge and screening attitudes in the population with lower EA remains to be investigated in a future effectiveness study, the LEAD trial (P Gabel, MD, unpublished data, April 2018). The DA will be provided to citizens as a link in a digital mail sent by a conventional mail to citizens who are exempt from digital communication. Hence, all eligible citizens will receive the link, regardless of their Internet accessibility or skills. Subject to such evaluation, this DA might guide decisions when developing information material for citizens with lower EA in other screening programs.

### Conclusions

The development of this DA identified the needs and preferences of citizens with lower EA regarding the level and amount of content in an eHealth solution for decision making about participation in CRC screening. The DA appeared acceptable and accessible for citizens with lower EA, enabling citizens to reflect on the benefits and harms of CRC screening to decide about screening participation.

## Appendix

Multimedia Appendix 1 Step 4a interview guide.

Multimedia Appendix 2 Step 4b interview guide.

Multimedia Appendix 3 Step 5 interview guide.

Multimedia Appendix 4 Step 6 interview guide.

## Abbreviations

CRC: colorectal cancer
DA: decision aid
EA: educational attainment
FIT: fecal immunochemical test
gFOBT: guaiac fecal occult blood test
GP: general practitioner
IPDAS: International Patient Decision Aid Standard
