# Supplementary material for: Developing a Self-Administered Decision Aid for Fecal Immunochemical Test–Based Colorectal Cancer Screening Tailored to Citizens With Lower Educational Attainment: Qualitative Study
Source: JMIR Form Res. 2018 May 22;2(1):e9. doi: 10.2196/formative.9696 (PMC6334704; doi:10.2196/formative.9696)
Supplement: Multimedia Appendix 1 [file formative_v2i1e9_app1.pdf]

Appendix 1

Interview guide – Focus group (Step 4a)

Design

- Did you read the pop-ups?
- Did you see the "read more"-function?
- Did you use the "read more" function?
- Did you appreciate the opportunity the gain more detailed knowledge?
- Did you like the layout?
- Did the layout make sense to you?
- Did you experience any difficulties navigating the webpage?

Values clarification exercise

- How did you experience answering the questions?
- Did the questions make sense in the context of the presented information?

Choice indicator

- How do you like the choice indicator?
- Does it make sense to you?
- Is the indicator useful or useless?
- Did the questions make you consider whether or not YOU would consider taking up screening?

Pictograms

- Did you look at the pictograms?
- How did you understand the pictograms?
- Is it clear to you, that these persons represent the first twenty persons in a larger population of 100-1000 persons?
- How did you like the pictograms?
- How did you feel about guessing before seeing the answer?

Risk presentation

- Did you read the numbers?
- Did the numbers make sense to you?
- How did you like the absolute numbers?
- How did you like the relative numbers?
- In some cases proportions was out of 25 persons, and at other times proportions was out of 100 or 1000 persons. Which proportions did you best relate to or understand?

Appendix 1

Flow chart

- Did you look at the flow chart?
- How did you like the flow chart?
- What does the flow chart describe?
- Did you like the flow chart?
- Does the flow chart play an important role in the screening information?

Headings

- The heading: Bowel cancer screening – an offer for you
  - Does it cover the contents of the information material?
  - How do you feel about the wording?
  - What does the heading say about the sender of the message?
  - Do you feel any obligations as the receiver of this message?
- Were the subject headings sufficient?
- Did you know where to find the information you were looking for?
- Were the content and the subheadings clear?

Content

- How did you feel about the amount of information?
- Was the content relevant?
- Is there any other information you would need to make a decision about screening participation?

Usability

- If you were provided with this information material, would you use it?
- Would you recommend it to friends and family?
- How do you feel about the length of the information material?
- How do you feel about the figure/animation/text ratio?
