# Supplementary material for: Developing a Self-Administered Decision Aid for Fecal Immunochemical Test–Based Colorectal Cancer Screening Tailored to Citizens With Lower Educational Attainment: Qualitative Study
Source: JMIR Form Res. 2018 May 22;2(1):e9. doi: 10.2196/formative.9696 (PMC6334704; doi:10.2196/formative.9696)
Supplement: Multimedia Appendix 2 [file formative_v2i1e9_app2.pdf]

**Appendix 2**

# Questions for alpha testing (step 4b)

## Content

- Relevance
- Length

## Comprehensibility

- Amount of information
- Level of details

## Usability

- Navigation
- Opportunity to seek help and advice if needed

## Design

- Layout
- Colors
- Figure/animation/text ratio
