# Supplementary material for: Developing a Self-Administered Decision Aid for Fecal Immunochemical Test–Based Colorectal Cancer Screening Tailored to Citizens With Lower Educational Attainment: Qualitative Study
Source: JMIR Form Res. 2018 May 22;2(1):e9. doi: 10.2196/formative.9696 (PMC6334704; doi:10.2196/formative.9696)
Supplement: Multimedia Appendix 3 [file formative_v2i1e9_app3.pdf]

Appendix 3

Questions for peer review (step 5)

We would appreciate your general comments on the webpage. In particular regarding:

- Relevance
  - Is the information sufficient in order to make a decision about screening participation?
  - Is there too much/too little information?

- Usability
  - Is a webpage suitable?
  - Would you use (citizens)/recommend (healthcare professionals) a webpage for screening information purposes?

- Readability
  - Does the text make sense to the targeted population (50-74-year-olds)?
  - Are sentences too formal or sufficiently easy to understand?

- Design
  - Is the webpage easy to navigate?
  - Is it intuitive to use?
  - Are any functions problematic or ineffective?
