# Supplementary material for: Developing a Self-Administered Decision Aid for Fecal Immunochemical Test–Based Colorectal Cancer Screening Tailored to Citizens With Lower Educational Attainment: Qualitative Study
Source: JMIR Form Res. 2018 May 22;2(1):e9. doi: 10.2196/formative.9696 (PMC6334704; doi:10.2196/formative.9696)
Supplement: Multimedia Appendix 4 [file formative_v2i1e9_app4.pdf]

Appendix 4

Interview guide – User-testing (Step 6)

Telephone interview:

You have received link for an alternative information material, and I would like to ask you some questions, regarding this. Some questions, you will be able to answer with a "yes" or "no", while others are more reflective, and call upon deeper explanations and comments. You can state your own comments at any point during this conversation.

Firstly: Have you read the material?

- Yes
- No
  - Why not?
    - Didn't have the time
    - Didn't want to?
    - Technical problems
    - The link didn't work

	Yes	No	Don't know
1. Did you spend a long time reading the material?	☐	☐	☐
2. How much time do you think you spent? Your best guess?			
3. Did you read all of the information?	☐	☐	☐
4. If no in 3: Did you skip any text?	☐	☐	☐
5. If no in 3: Which text did you skip?			
6. If no in 3: How did you choose which text was relevant to you?			
7. Was it easy to seek help or guidance if you experienced difficulties?	☐	☐	☐
8. How did you like the questions at the bottom of each step?			
9. Did you consider benefits and harms as you read the material?	☐	☐	☐
10. Did the results page at the last step make sense?	☐	☐	☐
11. Had you decided about screening uptake before reading the material?	☐	☐	☐
12. Did the material help you decide about screening uptake?	☐	☐	☐
13. Comments:			
Now I would like some feedback as to whether or not you found the material useful.			
14. Were there any technical difficulties?	☐	☐	☐
15. If yes in 12: What difficulties?			
16. Did you find the design appealing?	☐	☐	☐
17. Was it easy to navigate around the webpage?	☐	☐	☐
18. Was it intuitive to you which buttons to press?	☐	☐	☐
19. Were any features difficult to use?	☐	☐	☐
20. If yes in 19: Which ones?			
21. Were there any unnecessary features?	☐	☐	☐
22. If yes in 21: Which ones?			
23. Could any extra or fewer features have increased the user-friendliness, in your opinion?	☐	☐	☐
24. If yes in 23: Which features?			
25. Comments:			

Appendix 4

The information material is available online. This can be a challenge to some, but at the same time regular updates with valid information is secured. Further, special features, such as the questions at each step, and the choice indicator at the end are a possibility. Lastly, targeting the material according to gender and age is possible with an online format.			
26. In general, do you find an online information material targeted at 50-74-year-olds appropriate?	☐	☐	☐
27. If no in 24: Why is it inappropriate?			
28. Considering the benefits, mentioned above, do you think they outweigh the shortcomings of an online information material?	☐	☐	☐
29. If no in 28: Why not?	☐	☐	☐
30. What format is most appropriate for information material to 50-74-year-olds, in you opinion?<br>Leaflets (printed)<br>Oral information<br>Online information<br>Audible information<br>Other			
31. If Other in 30: What format?			
32. A read-aloud feature for the pop-ups has been considered. How do you feel about this consideration?			
33. Would you use this link if it was provided in the mail with the invitation for screening, you received in your E-boks?	☐	☐	☐
34. Would you recommend this material to friends and family?	☐	☐	☐
35. If yes in 34: Do you know anyone, who would benefit from this material?			
36. Comments:			

That was all. Do you have any further comments?

Thank you for your time, we appreciate it.

Have a nice day.
